# Progress in Medicine

**Published:** 1898-11-12

**Authors:** 


### Progress in Medicine.

### DISEASES OF CHILDREN.

*(Continued from page 50.)*

**Congenital Hypertrophic Stenosis of the Pylorus in Infants.**—Dr. S. J. Meltzer[12] records a case of this affection, which was correctly diagnosed and surgically treated. During the first two weeks of life the ouly abnormal symptoms observed were restlessness, extreme thirst, and occasional vomiting. The last of these then increased in frequency, and continued until within a few days of death. This vomiting was always forcible; it occurred soon after drinking, and at no other time; the vomit never contained bile, and did not show signs of fermentation or a catarrhal condition of the stomach. When the infant was four weeks old the stomach was washed out, and on palpation the empty stomach could be felt as a small tumour situated high up in the epigastrium. At this stage the stomach usually showed active peristalsis when full, but gradually a state of atony developed, and it became more and more permanently dilated, and no movements could be detected. The vomiting became less marked; there was no defæcation, and but little urination; and the child commenced to lose weight. The condition having been diagnosed, a surgeon performed gastro-enterostomy by means of a Murphy's button of the smallest size he could obtain; but the infant died 30 hours later. From the autopsy the anatomical diagnosis was stenosis of the pylorus from fibrous hyperplasia of the submucosa, and hyperplasia of the inner muscular layer, especially of the proximal half of the pyloric portion of the stomach. The author is hopeful that surgical interference may yet prove successful in relieving these] cases, and suggests that pylorectomy would probably be the best operation.

**Fever Following Operations for Pyothorax.**—Three hundred cases have been analysed by Dr. Augustus Caillé[13] with reference to this question of pyrexia and its cause after operation for empyema. One case was due to the irritation of a lost drainage tube which was found in the thoracic cavity. Instances of a rise in temperature from intoxication with iodoform or carbolic acid were not rare. Secondary and extra-thoracic abscesses as a cause of fever were frequently met with. A group of cases was formed of those dependent on a distinct specific infection or contagion, such as measles, scarlatina, erysipelas, diphtheria, and specific vulvovaginitis. The possibility of an empyema occurring on the side not operated on must also be kept in mind. Unresolved pneumonia or pulmonary tuberculosis may cause persistent pyrexia, a diagnostic point between these two being that the excursions of the mercury are much greater in the latter than in the former. In cases of tuberculosis of the lung with pulmonary abscess of long standing and secondary empyema, there is usually no satisfactory drop in the temperature after operation. Acute septic empyema in young infants is usually accompanied by a high temperature after operation, and, even with good drainage and good lung expansion, the most frequent termination is in death.

**The Treatment of Pott's Disease.**—Dr. Perier[14] outlines the following treatment: (1) Upon the first manifestation of the disease the child should be put to bed, on a horsehair mattress, and kept in the horizontal posture. (2) It should, without change in posture, be kept as much as possible in the sunshine, either on land or water. (3) In winter the child receives before breakfast one-half to one tablespoonful of cod-liver oil, occasionally the syrup of tannic iodide. (4) Before each of the two chief meals the patient receives, during the first two weeks, from one to four drops of Fowler's solution; during the two succeeding weeks a teaspoonful to a tablespoonful of the syrup of the iodide of iron. (5) The skin functions are to be improved and kept active by means of baths, massage, &c. (6) The diet should consist of meat, eggs, meat powders, and the like; if the digestion is good, and in older children, vegetables of all sorts should be given.

**Congenital Facial Paralysis.**—Two cases of this affection are recorded by Dr. H. M. Thomas,[15] the patients being brothers, aged respectively 21 and 19 years. The history showed that the lesion had been present since birth. In earliest infancy the under lip was noticed to droop, the eyes did not close properly, and the face remained expressionless. When they came under observation the condition was practically the same in both. The face was expressionless, the forehead smooth, the eyes wide open, the mouth open, and the lower lip was large and everted. The labials could not be properly pronounced owing to non-closure of the lips. The muscles of mastication acted well and equally on the two sides. Sensation in the face was normal, and the sense of taste was unaffected. The movements of the eyeballs were normal, but the patient was entirely unable to raise or contract the eyebrows. When trying to close his eyes the eyeballs were rolled up, and the upper lids were somewhat relaxed. He was unable to elevate his upper lips or to pucker his mouth, or, indeed, to close it. The only oral movement possible was a slight drawing outwards and downwards by the action of the platysmæ muscles. In connection with this congenital facial diplegia, the author points out that not infrequently one or more muscles of the eyeball are affected. Out of 14 cases with this association the defect in the seventh nerve was combined with a similar lesion in the sixth nerve no less than twelve times. This frequent association leads the author to believe that mal-development in the medulla, near the origin of these two nerves, is a more probable explanation of the condition than a congenital muscular defect.

**Laparotomy in Tuberculous Peritonitis.** — Three varieties of this affection are described by Professor Monti.[16] In the first class, are placed those cases, subacute in character, with inflammation of the peritoneum, accompanied by a serous exudation; in the second class, those with the mesenteric glands in a state of cheesy degeneration and intestinal matting together; in the third, those of intestinal matting without any special change in the mesenteric glands, but with palpable thickenings. He has observed 21 cases of tuberculous peritonitis, of which 10 were treated by laparotomy and 11 by medical measures only. Of the former seven were cured and three died; of the latter two were cured and the remainder were improved to the extent that all acute symptoms had subsided when they passed from observation. He concludes that tuberculous peritonitis with exudation can be cured by medical treatment, but less frequently and more slowly than by laparotomy. In the absence of fluid, and more especially if there is much glandular enlargement, laparotomy is not to be advised.

[12] Med. Rec., Aug. 20, 1898. [13] Arch. of Pediat., Aug., 1898. [14] Med. Rec., Sept. 3, 1898; and Jour de Med. de Paris, No. 5, 1897. [15] Jour. Nerv. and Ment. Dis., Aug., 1898. [16] Arch. f. Kinderheilk, Bd. xxiv., Heft 1 and 2.

### DISEASES OF DIGESTIVE ORGANS.

(*Concluded from page* 67.)

**Effects of Intestinal Irrigation.**—Coleman Kemp[52] lays down (1) that if we do not wish to raise the pulse tension the temperature of the injection should be 100 deg. to 103 deg.; when we do wish to raise it and to stimulate the heart, as in shock, the temperature should commence at 110 deg., and rise steadily to 120 deg. Cold is stimulant at first, but soon causes depression. In shock from hæmorrhage the temperature should be 110 deg. to 120 deg., with a double current enema. Hot injections raise the temperature of the body, and still more that of the blood. Cold irrigations have been successfully used to reduce temperature in dysentery and infantile diarrhœa. In kidney disease irrigations at 110 deg. to 120 deg. stimulate the kidney circulation, and if continued for twenty minutes, aid by the absorption of fluid from the intestines. These hot injections are of great value in uræmia, and insufficient action of the kidneys, Finally, he notices that absorption from the large intestines takes place in twenty minutes, but cold prevents this absorption altogether, and cold injections should therefore be forbidden in renal disease. Brandenburg and Hupperz[53] think that rectal nutrient enemata should be more frequently employed, and find that casein and fats are badly absorbed. They have tried Alcarnose, a mixture of albumoses and maltose, without any unpleasant effects. The nitrogen excretion in the urine was greatly increased, so that sometimes half the nitrogen in the clyster was thus obtained, and there was no increase in the intestinal putrefaction. They came to the conclusion that Alcarnose was a valuable rectal nutriment. Bardet,[54] in reviewing the question of predigested food, also lays stress on the value of albumoses in preference to peptones, as more nutritious and less likely to contain toxic substances. He praises Somatose, which contains 10 per cent. of nitrogen. An increase of weight can be obtained on a diet of albumose if a certain amount of hydrocarbon is added, which does not appear to be the case when parapeptone is substituted for albumose.

**Intestinal Auto-Intoxications.**—These differ according to Müller[55] from the intoxications of anæmia, thyroidism, and gout, inasmuch as they are caused by saprophytes in the intestinal canal. Though very few exact facts are known, poisoning of this kind does occur, especially in children, and where obstruction of the bowel has occurred. He thinks the urotoxic coefficient, of which so much has been heard of late, is untrustworthy. Intestinal antisepsis appears to be impossible. Brieger agrees with him that purgatives and emetics are the best mode of treatment. Boas holds that a diuretic should be also used, and remarks that calomel is both diuretic and purgative. Gilbert and Galbrun[56] report from their experiments that benzonaphthol will reduce the number of microbes in the fæces about 55 per cent. A daily enumeration fo 27 days was made upon the fæces of a healthy in dividual, to whom they gave 3·5 grammes to 4 grammes of benzonapthol daily.

[52] Amer. Jour. Med. Sci. April. [53] Brit. Med. Jour., June 11. [54] Bulletin de Therap., Mar 30. [55] Lancet, May 21. [56] La Semaine Med., May 11.

